# The efficiency of arbuscular mycorrhiza in increasing tolerance of *Triticum aestivum* L. to alkaline stress

**DOI:** 10.1186/s12870-022-03790-8

**Published:** 2022-10-17

**Authors:** Fatma Aly Farghaly, Nivien Allam Nafady, Dalia Ahmed Abdel-Wahab

**Affiliations:** 1 Botany and Microbiology Department, Faculty of Science, Assiut University, Assiut, 71516 Egypt; 2 Botany and Microbiology Department, Faculty of Science, New Valley University, El Kharja, Egypt

**Keywords:** Alkalinity, *Triticum aestivum*, Arbuscular mycorrhiza, Lipid peroxidation, Antioxidant system, Mineral nutrition

## Abstract

**Background:**

Evaluation of native soil microbes is a realistic way to develop bio-agents for ecological restoration. Soil alkalinity, which has a high pH, is one of the most common concerns in dry and semi-arid climates. Alkaline soils face problems due to poor physical properties, which affect plant growth and crop production. A pot experiment was carried out to investigate the impact of native mycorrhizal fungi (AMF) on the wheat plant (*Triticum aestivum* L.) under two levels of alkalinity stress -T1 (37 mM NaHCO_3_), T2 (74 mM NaHCO_3_) - at two developmental stages (the vegetative and productive stages).

**Results:**

Alkalinity stress significantly inhibited the germination percentage, plant biomass, photosynthetic pigments, and some nutrients (K, N, and P). Mycorrhizal inoculation improved growth parameters and productivity of wheat-stressed plants. However, lipid peroxidation was significantly lowered in mycorrhizal-inoculated plants compared to non-inoculated plants. Catalase and peroxidase were inhibited in wheat leaves and roots by alkalinity, while mycorrhiza promoted the activity of these enzymes.

**Conclusion:**

The results of this study demonstrated that alkalinity stress had highly negative effects on some growth parameters of the wheat plant, while AMF inoculation attenuated these detrimental effects of alkalinity stress at two stages by reducing the pH and Na concentration and increasing the availability of P and the productivity of wheat in particular crop yield parameters.

## Background

Wheat is the major essential crop in Egypt and is reported as a strategic commodity, which is considered the main ingredient in the Egyptian diet [1]. Expansion of wheat production in Egypt is necessary to supply the demands of a rapidly growing population and reduce dependence on importing wheat [2]. Some studies deal with the effect of salinity-alkalinity stress [3] and sodic stress [4] on wheat plants at different stages.

  Soil salinization and alkalinization have become big contributions to the global trend of land degradation, particularly in arid and semi-arid regions [5]. Soil salinity or alkalinity affects around 900 million hectares of soil resources globally, accounting for 70% of all agricultural land [6, 7]. Alkalinity is linked to the presence of NaHCO_3_ or Na_2_CO_3,_ which raises the pH level further due to salt stress. Alkalinity, according to many studies, is more harmful than salinity stress [3, 8]. The high pH level causes ion imbalances and metabolic disruptions, as well as endosperm toxicity, which is the source of supply for early seedlings [3]. Also, the high pH level that correlated with a large quantity of adsorbed Na^+^ represses the availability of several essential nutrients, especially iron, phosphorus, and manganese in alkali soils [9]. Under salinity-alkalinity stress, plants produce reactive oxygen species (ROS) [10]. Reactive oxygen species at high concentrations impair cell membranes and disrupt a wide range of important macromolecules, including photosynthetic pigments and proteins [11].

  The production of enzymatic substances such as catalase (CAT), ascorbate peroxidase (APX), superoxide dismutase (SOD), and guaiacol peroxidase (GP) has been reported to eliminate excess ROS produced in plants under salinity stress [12]. Likewise, one of the most important biochemical responses of plants to abiotic stresses is the overproduction of various forms of compatible solutes such as proline, sugars, soluble proteins, amino acids, etc. [13].

  Members of the phylum Glomeromycota form an arbuscular mycorrhizal association with most of the plants and provide extrinsic protection against various stresses such as salinity and alkalinity [14, 15]. Mycorrhiza fungi are natural root symbionts and are generally known as bio-fertilizers. Such endosymbionts play a vital role in the functioning of the ecosystem and sustainable crop improvement [16].

It was documented that arbuscular mycorrhiza fungi (AMF) increased plant growth parameters [17] as well as the uptake of multiple important elements like nitrogen and phosphorus by stressed plants [16]. This growth promotion is linked to the fact that AMF extends the absorbing network beyond the nutrient depletion zones of the rhizosphere, allowing access to a larger volume of soil and also modifying the level and distribution of nutrients within wheat and barley grains [18]. Fungal hyphae are also noticeably thinner than roots, allowing them to enter small pores and absorb more nutrients [19]. Mycorrhiza fungi can alleviate stressful situations by enhancing their ability to scavenge reactive oxygen species (ROS) [20].

Previous studies have reported the mitigated effects of AMF symbioses under salinity-alkalinity conditions on wheat and soybean plants [21, 22]. It was also observed that inoculating salt-stressed watermelon with *Funneliformis mosseae* minimized oxidative damage by enhancing antioxidant enzyme gene expression [6]. Inoculation with *Acaulospora laevis*, and *Gigaspora margarita* increased rice plant yield by 125% and 143% as compared with the non-inoculated controls, at 75 and 120 mM salt levels, respectively [23]. Mycorrhizal native inoculum (*Pacispora franciscana*, *Funneliformis mosseae*, *Funneliformis geosporum*, *Rhizophagus irregularis* and *Glomus tenebrosum*) can improve water tolerance, leading to higher barley grain yield and quality [24].

The Egyptian soil is generally characterized as being slightly alkaline which is mainly due to the dry environment [25]. In this context, reclaimed alkaline soil can provide an additional cultivable area for supporting the production of wheat. The useful concept in agriculture is highlighted in the reduction of chemical additives to ameliorate alkalinity stress, which causes a reduction in growth and productivity stages by using the beneficial soil-dwelling fungi that exhibit symbiotic association with plants such as mycorrhizal fungi. As a result, we meet the following objectives in this study: 1) investigate the symbiotic interaction between wheat and AMF to counteract the negative impacts of alkalinity stress, and 2) study the effects of alkalinity stress on wheat growth, photosynthetic pigments, some physiological indices, antioxidant enzymes, some minerals concentration and yields of greenhouse-grown wheat plants.

## Materials and methods

### Soil physicochemical analysis

The soil was bulked to give a composite sample. The soil was air-dried and sieved to ~ 2 mm. Then the chemical and physical characteristics of the used soil were determined before planting and during the cropping season in the soil–water extract (1:5 w/v). Soil electrical conductivity (EC), and pH were measured according to [26]. Soil sodium (Na^+^) and potassium (K^+^) were determined using a Carl Zeiss flame photometer, whereas the concentrations of P and N were determined spectrophotometrically according to [27]. Organic matter (OM%) concentration was estimated by losing in the ignition at 600 °C for 3 hrs. according to [28]. Bicarbonates were estimated according to the method described by [29]. The soil texture was loam (20.3% clay, 33.7% silt and 46% sand) with pH 7.25, EC_e_3.5 dSm^−1^, organic matter (OM) 1.76%,183 mg Kg^−1^ HCO_3_^−^, 767.5 mg Kg^−1^ Na^+^, 37.8 mg Kg^−1^ K^+^, 128.75 mg Kg^−1^ available P and 128.75 mg Kg^−1^available N.

### Mycorrhizal inoculum

The arbuscular mycorrhizal fungi (AMF) were recovered from the rhizosphere trap culture grown with *Zea mays* L. as a host plant for 12 weeks in a greenhouse by the wet sieving method [30]. The spores were previously recovered from alkaline soil [25]. The mycorrhizal fungi were *Acaulospora laevis*, *Funneliformis geosporum*, *Funneliformis mosseae*, and *Cetraspora armeniaca*. These genera are identified morphologically according to [31] and [32] and allow the classification of [33]. The mycorrhizal inoculum consists of infected root segments, extradical hyphae, rhizosphere soil, and spores as inoculum for pot experiments. Fifty grams of AMF containing approximately 90–100 spores/10 g soil were placed at 3–5 cm depth directly before wheat planting to promote fungal inoculation of plant roots. Non-fungal treatments accepted an equal mass of autoclaved growth. Pot experiments were randomly divided into two major groups: non-mycorrhizal and mycorrhizal.

### Plant culture and experimental conditions

Wheat caryopses (*Triticum aestivum* L. cv Gemmeza10) were kindly supplied by the Agricultural Research Center, Giza, Egypt. This cultivar is most widely cultivated in the Nile Valley area. Caryopses were surface sterilized by 70% ethanol (30 s), followed by 5% NaOCl (10 min), and washed four times with sterilized distilled water [34]. Twenty caryopses of well-selected wheat were planted in plastic pots on the soil. Each pot was filled with 2.5 kg of soil that contained a mix of sieved air-dried clay and sand soil (2: 1 v). The study is conducted with three alkaline treatments (T0, T1 & T2) and mycorrhizal treatments (AMF + T0, AMF + T1 and AMF + T2). Three levels as control = 0 gm NaHCO_3_ / 2.5 kg; pH 7.25, T1 = 5 g NaHCO_3_ / 2.5 kg ≈ 37 mM; pH 8.68 and T2 = 10 g NaHCO_3_ / 2.5 kg ≈ 74 mM; pH 9.14 were tested that selected from preliminary experiments (data not shown). The experiment was conducted in the greenhouse of the Botany and Microbiology Department, Faculty of Science, Assiut University, during the growing season of wheat under a natural temperature (23–29 °C), light (11-hrs photoperiod), and 48–52% humidity conditions. Plants were grown at a soil water potential of field capacity. We did not add any fertilizers to the used soil.

### Parameters studied

#### The germination percentage of caryopses

Seedling emergence was recorded periodically on the 6^th^, 8^th^, 10^th^, and 15^th^ days. It is defined as an emergency when plumules were completely exposed. The percentage of germination (GR %) was calculated using the following equation [35]:

$$\mathbf G\mathbf R\left(\boldsymbol\%\right)=\frac{\mathbf N\mathbf u\mathbf m\mathbf b\mathbf e\mathbf r\boldsymbol\;\mathbf{of}\boldsymbol\;\mathbf g\mathbf e\mathbf r\mathbf m\mathbf i\mathbf n\mathbf a\mathbf t\mathbf i\mathbf n\mathbf g\boldsymbol\;\mathbf c\mathbf a\mathbf r\mathbf y\mathbf o\mathbf p\mathbf s\mathbf e\mathbf s\boldsymbol\;\mathbf o\mathbf f\boldsymbol\;\mathbf e\mathbf a\mathbf c\mathbf h\boldsymbol\;\mathbf{tr}\mathbf e\mathbf a\mathbf t\mathbf m\mathbf e\mathbf n\mathbf t}{\mathbf T\mathbf o\mathbf t\mathbf a\mathbf l\boldsymbol\;\mathbf n\mathbf u\mathbf m\mathbf b\mathbf e\mathbf r\boldsymbol\;\mathbf o\mathbf f\boldsymbol\;\mathbf{so}\mathbf w\mathbf n\boldsymbol\;\mathbf c\mathbf a\mathbf r\mathbf y\mathbf o\mathbf p\mathbf s\mathbf e\mathbf s}\times100$$

### Vegetative growth

All plants were regularly irrigated with tap water as needed to preserve soil moisture near field capacity. After 56 days, some of the wheat plants were gently removed from the pots, washed with water, separated into shoots, roots, and leaves, then rapidly frozen in liquid nitrogen and stored at -80°C for biochemical analysis. Fresh weight (FW) of roots and shoots was taken instantly after harvesting, whereas dry weight (DW) was determined after drying the plant samples at 70°C for 72 hrs.

### Photosynthetic pigments

The photosynthetic pigments (Chlorophyll a, chlorophyll b, and carotenoids) were estimated separately using the spectrophotometric method (Unico UV-2100 spectrophotometer) recommended by [36]. Chlorophyll a and b concentrations were calculated mathematically as total chlorophylls and carotenoids concentration was calculated as mg/g FW.

### The plant osmolyte metabolites

Proline concentration was determined in the leaves and roots of the wheat plant following the acid-ninhydrin technique [37]. It was measured spectrophotometrically at 520 nm with toluene as a blank and expressed as mg/g FW. The soluble protein, soluble sugars, and total free amino acids in leaves and roots extract of the wheat plant were measured according to the method described by [38] and expressed as mg/g DW.

### Lipid peroxidation

Lipid peroxidation was assessed in the leaves and roots to evaluate membrane damage. Therefore, MDA was measured using the thiobarbituric acid (TBA) test. Lipid peroxidation was calculated using an extinction coefficient (155 mM^-1^ cm^-1^) and expressed as µM/g FW [39].

### Enzymes assay

Frozen wheat leaves and roots were homogenized in liquid nitrogen in 5ml of 100 mM phosphate buffer, pH 7.8 containing 0.1 mM ethylenediaminetetraacetic acid (EDTA) and 0.1 g polyvinylpyrrolidone (PVP). After centrifugation at 12000 rpm for 10 min at 4º C, the supernatant was utilized to assay the activities of Lipooxygenase (LOX, EC 1.13.11.12), Catalase (CAT, 1.11.1.6), Peroxidase (POD, EC 1.11.1.7) by using a UV visible Unico UV-2100 spectrophotometer. Following the method of [40], LOX activity was assayed at 234 nm and expressed as DA_234_ mg protein^-1^ min^-1^. The CAT activity was evaluated one minute after H_2_O_2_ consumption using the [41] protocol and represented as DA_240_ mg protein^-1^ min^-1^. Using the method described by [42], POD activity was recorded at 470 nm and expressed as DA_470_ mg protein^-1^ min^-1^.

### Phenolic compounds concentration

The total concentration of phenolic compounds in wheat leaves was evaluated by the phenol reagent technique of Folin-Ciocalteu and expressed as mg/g FW [43]*.*

### Mineral composition

The dry samples of shoots and roots (0.03 g) were digested in 5 mL of conc. sulphuric acid, one ml sulphuric-perchloric mixture (1: 1 by volume) of 50% sulphuric and 30% perchloric and diluted with distilled water to 30 ml [44]. The concentrations of Na^+^, K^+^, P, and N were assayed as mentioned previously in soil analysis.

### Caryopses yield and its components

After 5 months of cultivation, some yield indexes such as spike number/plant, spikelet number/spike, caryopses number/spike, and weight of caryopses/spike were determined on three plants for each replicate for all treatments of the wheat plant.

### Mycorrhizal colonization levels

Mycorrhizal colonization of tested wheat roots was assessed after harvesting. Fresh wheat root segments (1.5 cm long pieces) were washed several times with sterilized water and cleared with KOH (10%) according to the staining protocol [45]. The gridline to intersect method was used to calculate mycorrhizal colonization parameters [46].

### Statistical analysis

The one-way ANOVA test was conducted and several comparisons were performed using the statistical package SPSS version 22.0 (SPSS, Chicago, IL) and following Tukey’s test. Principal component analysis (PCA) accounted for all the measurements of the different treatments using PAST (PAleontological STatistics) version 2.11.

## Results

### The percentage of germination

**Fig. 1:**
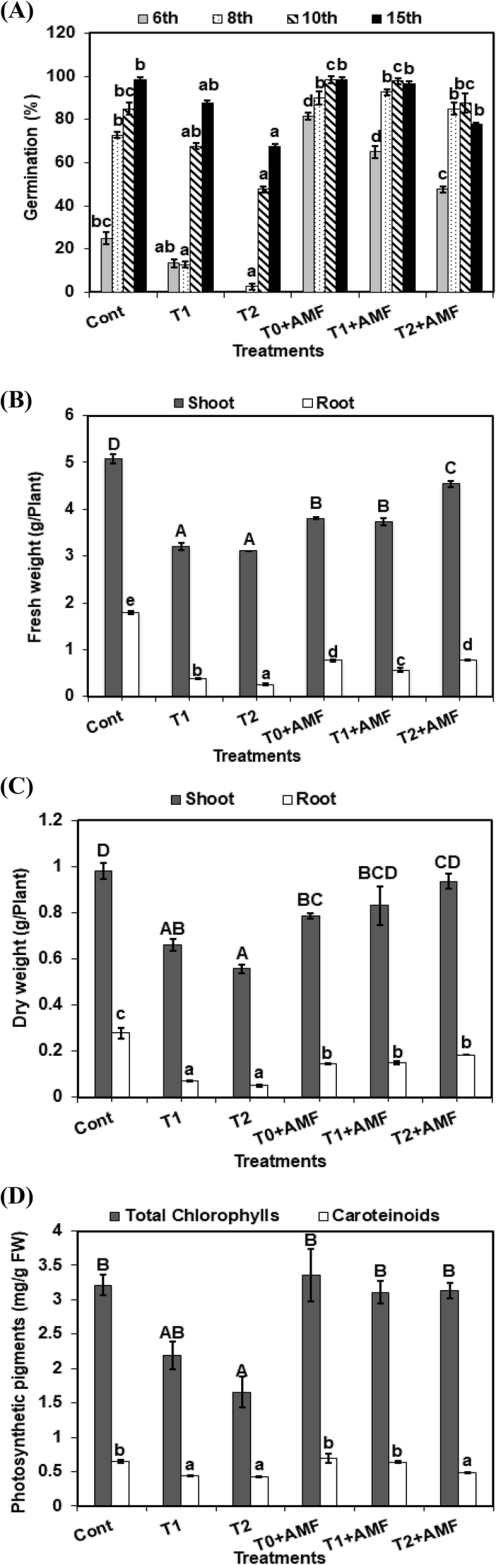
Impacts of mycorrhiza inoculation (AMF) and alkalinity levels (T1, T2) either under stressed and unstressed conditions on the germination percentage (**A**) at 6^th^, 8^th^, 10^th^, and 15^th^ (the days of germination), fresh weight (**B**) dry weight (**C**), and photosynthetic pigments (**D**) in the wheat plant. The data are means ± SD (*n* = 3). Different letters indicate statistically significant differences according to Tukey’s test (*p* ≤ 0.05)

The percentage of germination gradually increased with the emergence of seedlings per day (Fig. 1A). The control recorded the highest percentage of germination in wheat caryopses, whilst the two levels of alkalinity (T1 and T2) significantly reduced the germination percentage compared with the control seedlings. AMF inoculation increased the germination percentage of seedlings grown under non-stressed and stressed conditions.

### Growth parameters

The fresh and dry weight of shoots and roots significantly declined with the two levels of alkalinity (T1, T2). However, AMF inoculation significantly improved plant fresh and dry biomass and alleviated the negative effect of alkalinity stress (Fig. 1B, C).

The data in Fig. 1D showed that the synthesis of total chlorophyll fractions (Chl. a and Chl. b) and carotenoids in wheat leaves, cultivated in alkaline soils in T1 were reduced by 31.9%, 32%, and in T2 by 48.5%, 34.45% for total chlorophylls and carotenoids, respectively, compared with control plants, while AMF inoculation increased the synthesis of total chlorophylls by 28.67% and 46.7% in T1+AMF and T2+AMF, respectively and carotenoides by 8.84% and 30% at T2+AMF and T1+AMF, repectively compared to stressed plants in T1 and T2.

### The plant metabolism and osmolyte compounds

**Fig. 2:**
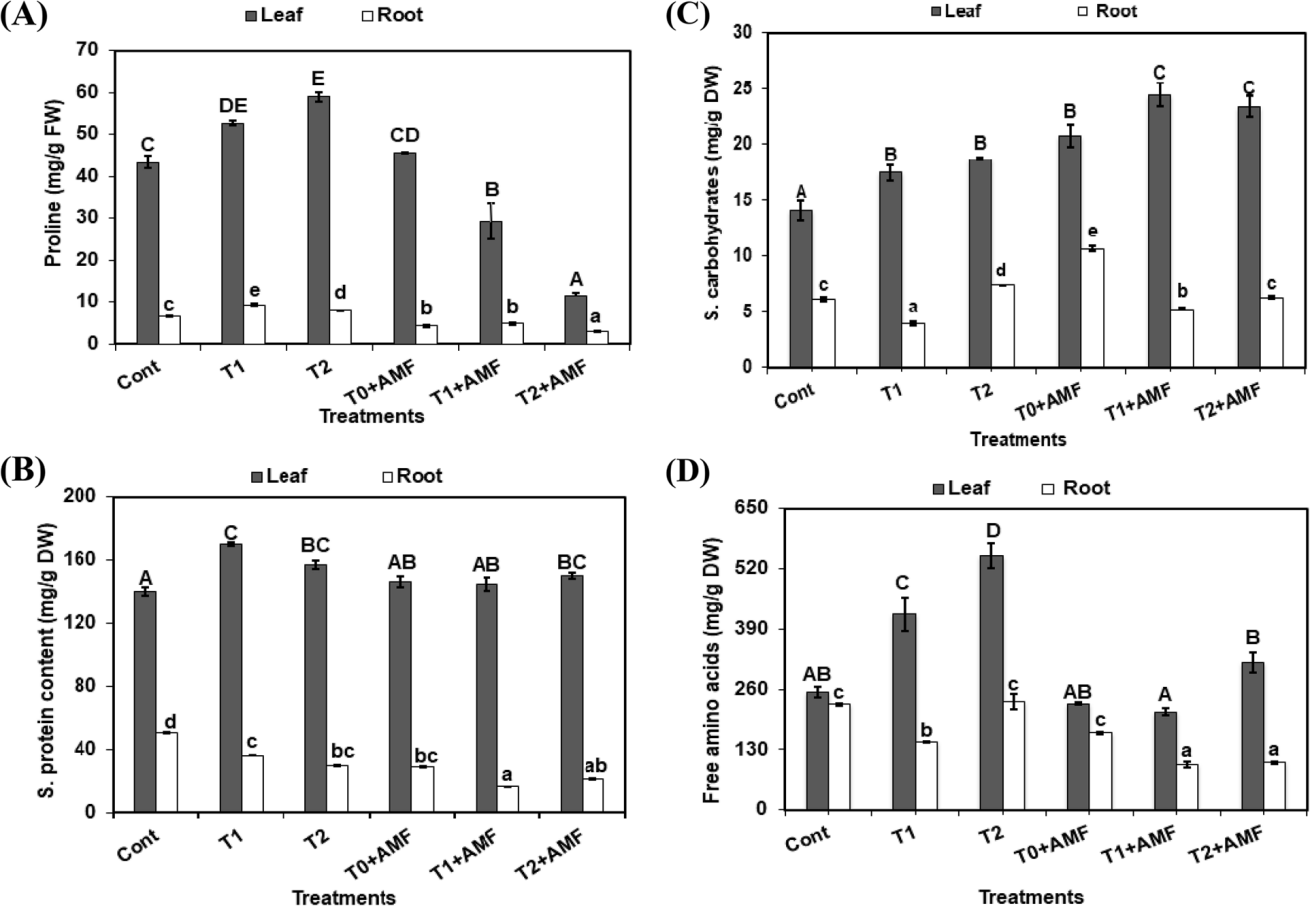
Impacts of mycorrhiza inoculation (AMF) and alkalinity levels (T1, T2) either under stressed and unstressed conditions on proline concentration (**A**), soluble protein concentration (**B**), soluble carbohydrates concentration (**C**), and free amino acids concentration (**D**) in the wheat plant. The data are means ± SD (*n* = 3). Different letters, capital for leaves, and small for roots indicate statistically significant differences according to Tukey’s test (*p* ≤ 0.05)

Alkalinity increased proline accumulation in wheat leaves and roots by about 21%, 40% in T1 and 35%, 21% in T2 over the control plants (Fig. 2A), while proline accumulation was dramatically reduced in plants growing on alkaline soils after AMF inoculation (T1 + AMF and T2 + AMF).

The concentration of soluble protein in leaves increased significantly with alkalinity (T1, T2). While AMF inoculation caused no significant changes in protein concentration of leaves in plants grown under unstressed (T0 + AMF) and stressed conditions (T2 + AMF, T1 + AMF) exhibited a significant decrease compared with T1. However, soluble protein in roots exhibited a significant reduction in all treatments without or with AMF inoculation (Fig. 2B).

Results showed that soluble carbohydrates were promoted significantly in all treatments in leaves and roots except T1 which diminished their concentration in roots compared with the control plants, whereas inoculation with AMF increased leaves soluble carbohydrate concentration in T1 + AMF, and T2 + AMF, which recorded 73.5% and 66.3%, respectively, over the control plants. Similarly, AMF inoculation under unstressed conditions (T0 + AMF) recorded the highest value of soluble carbohydrate concentration in roots, while, under stressed conditions, their concentration in roots exhibited a significant reduction in T1 + AMF compared with the control plants (Fig. 2C).

The free amino acid concentration of stressed plants in the leaves and roots was higher than AMF inoculated ones as shown in Fig. 2D. The free amino acids in leaves increased significantly with increasing alkalinity in stressed plants which recorded 66% at T1 and 116% in T2 over the control plants. On the other hand, in roots T1 inhibited the accumulation of free amino acids by 35.4% in comparison with the control plants.

It was observed that the highest level of alkalinity with the AMF inoculation in T2 + AMF enhanced free amino acids concentration in leaves by 25% over the control plants, whereas, in roots, the mycorrhiza inoculation under unstressed and stressed conditions had negative effects on their concentrations (T0 + AMF, T1 + AMF, and T2 + AMF) which decreased by 27%, 57%, and 55%, respectively, compared with the control plants (Fig. 2D).

### Lipid peroxidation

**Fig. 3:**
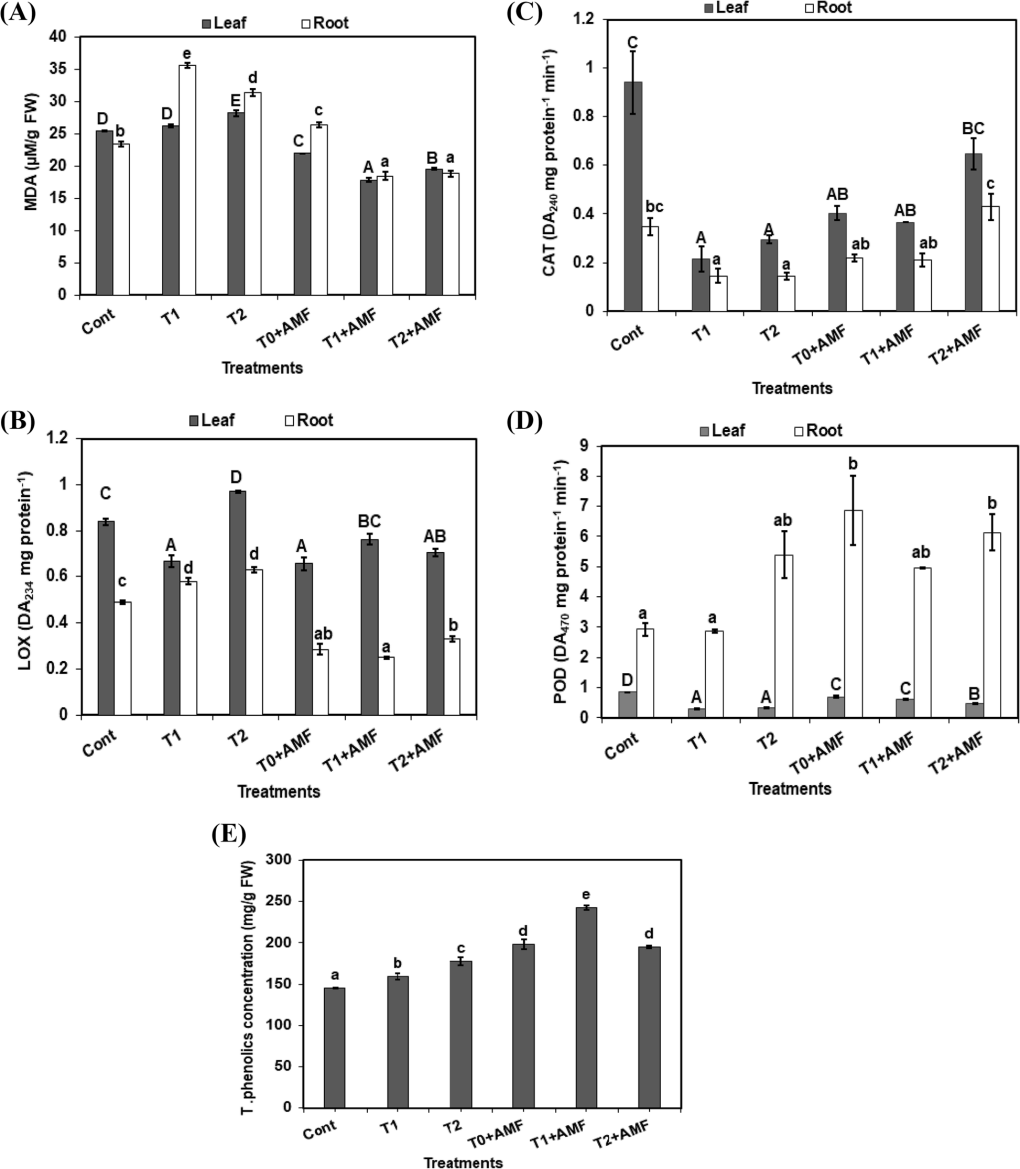
Impact of mycorrhiza inoculation (AMF) and alkalinity levels (T1, T2) either under stressed and unstressed conditions on lipid peroxidation; MDA (**A**), lipoxygenase; LOX (**B**), catalase activity; CAT (**C**), peroxidase activity; POD (**D**), and total phenolics concentration (**E**) in wheat plant. The data are means ± SD (*n* = 3). Different letters, capital for leaves, and small for roots indicate statistically significant differences according to Tukey’s test (*p* ≤ 0.05)

Alkalinity increased MDA accumulation in the leaves, especially in T2 which recorded 10% over the control plants, whereas their accumulation in roots was more enhanced under the same conditions which recorded 52.1% for T1 and 34.3% for T2 over the control plants. AMF inoculation significantly decreased lipid peroxidation of the leaves and roots in unstressed and stressed plants compared with the control ones while MDA exhibited significant stimulation in T0 + AMF by 12% over the control plants (Fig. 3A).

### Enzymes

Alkalinity stress significantly promoted the LOX activity in both leaves and roots of plants, except in leaves treated with T1 where it was inhibited by 22.8% compared with the control plants. Generally, inoculation with mycorrhiza decreased the LOX activity in leaves and roots of plants grown under stressed or unstressed conditions (Fig. 3B).

In wheat leaves or roots catalase activity (CAT) was markedly decreased by soil alkalization at both levels compared with the control plants. CAT activity was higher in mycorrhizal plants (both T0+AMF, T1+AMF and T2+AMF) compared with stressed non-mycorrhizal plants (T1, T2) (Fig 3C).

Compared to the control plants, peroxidase activity was significantly reduced in leaves in all treatments, but in roots, it was mostly enhanced. However, AMF inoculation promoted POD activity in most cases under alkalinized or unstressed soils (Fig. 3D).

### Total phenolics concentration

The highest level of alkalinity (T2) significantly increased phenolic concentration by 22 % over the control plants. Inoculation with AMF under stressed conditions (T1+AMF, and T2+AMF) resulted in a highly significant increase in phenolic concentration by 57.3%, and 11.6% respectively, over stressed plants (Fig. 3E).

### Caryopses yield parameters

Spike number/plant has a non-significant effect with increasing alkalinity levels, whereas inoculated plants with AMF under stressed conditions (T1 + AMF, and T2 + AMF) exhibited a significant increment of 57.5%, and 31.5%, respectively, over the control plants (Table 1). Similarly, alkalinity had a slightly negative effect on spikelet number/spike, which decreased as alkalinity increased in T1 and T2 compared to the control plants, while inoculation with AMF under stressed conditions (T1 + AMF, T2 + AMF) had no discernible effect (Table 1).

**Table 1:** Impact of mycorrhiza inoculation (AMF) and different alkalinity levels (T1, T2) either under stressed and unstressed on caryopses yield parameters of the wheat plant at productivity stage. The data are means ± SD (*n* = 3). Different letters indicate statistically significant differences according to Tukey’s test (*p* ≤ 0.05)

Treatments	Spike number/plant	Spikelet number/spike	Caryopses number/spike	Wt. caryopses/spike
**Cont**	7.3 ± 0.33^ab^	18 ± 0.30^bc^	32 ± 1.2^c^	0.73 ± 0.07^b^
**T1**	6.7 ± 0.40^a^	17 ± 0.14^a^	21 ± 2.1^ab^	0.38 ± 0.009^a^
**T2**	6.0 ± 0.57^a^	17 ± 0.30^ab^	16 ± 0.21^a^	0.41 ± 0.005^a^
**T0 + AMF**	8.0 ± 0.57^ab^	19 ± 0.30^c^	28 ± 0.3^bc^	0.57 ± 0.04^ab^
**T1 + AMF**	11.5 ± 0.86^c^	18 ± 0.30^bc^	24 ± 2.3^ab^	0.60 ± 0.11^ab^
**T2 + AMF**	9.6 ± 0.33^bc^	18 ± 0.30^bc^	24 ± 2.9^ab^	0.58 ± 0.08^ab^

  All levels of alkalinity stress had strong inhibitory effects on caryopses number/spike, which decreased significantly with rising alkalinity stress and recorded 65.6% and 50% in T1 and T2, respectively, compared to the control plants. Inoculated wheat plants with AMF under unstressed conditions had no significant effect on caryopses. While the previous condition under both alkalinity levels (T1 + AMF and T2 + AMF) suppressed the number of caryopses by 75% compared to the control plants (Table 1).

Weight of caryopses/spike was inhibited gradually and markedly by 47% and 43% in T1 and T2, respectively with increasing alkalinity levels, when compared with control plants. However, AMF inoculation improved its under-stressed conditions (T1 + AMF and T2 + AMF) by 26%, and 22.7%, respectively, over the stressed plants (Table 1).

### Physical and chemical soil properties

The physical and chemical properties of the soil used throughout this study were determined before planting and during the cropping season. It was observed that soil EC values increased significantly only at the highest level of alkalinity during the cropping season. Inoculation with AMF under un-stressed conditions (T0 + AMF) significantly decreased the EC values while having no significant change with other treatments (Table 2).

**Table 2:** Impact of mycorrhiza inoculation (AMF) and different alkalinity levels (T1, T2) either under stressed and unstressed on physical and chemical properties of soil. The data are means ± SD (*n* = 3). Different letters indicate statistically significant differences according to Tukey’s test (*p* ≤ 0.05)

Treatments	EC	pH	Na^+^	K^+^	available P	N
	**(dSm**^**−1**^**)**			**Concentrations (mg/Kg)**		
**Cont**	3.5 ± 0.15^b^	7.25 ± 0.0^a^	767.5 ± 15.87^a^	37.8 ± 0.04^d^	128.7 ± 15.1^a^	140 ± 5.8^c^
**T1**	3.6 ± 0.01^b^	8.68 ± 0.0^c^	1042.5 ± 12.99^c^	24.0 ± 0.11^b^	370.0 ± 31.7^b^	130 ± 2.9^c^
**T2**	4.8 ± 0.10^c^	9.14 ± 0.02^d^	1162.5 ± 24.5^d^	33.6 ± 1.50^c^	817.5 ± 18.8^e^	65 ± 5.7^a^
**T0 + AMF**	3.1 ± 0.09^a^	7.6 ± 0.04^b^	760 ± 2.88^a^	23.4 ± 0.35^b^	650.0 ± 57.7^d^	70 ± 0.0^a^
**T1 + AMF**	3.7 ± 0.09^b^	7.5 ± 0.05^b^	982.5 ± 7.21^b^	20.1 ± 0.57^a^	557.5 ± 7.21^c^	112.5 ± 4.3^b^
**T2 + AMF**	3.7 ± 0.03^b^	7.75 ± 0.04^c^	1117.5 ± 1.44^d^	20.1 ± 0.16^a^	442.5 ± 50.5^bc^	170 ± 5.8^d^

By treatment of the soil with 37 mM (T1) and 74 mM (T2) NaHCO_3_, their pH values significantly increased to 8.68 and 9.14, respectively. However, the addition of AMF inoculum into these soils mitigated the alkalinity to be around the neutral conditions (Table 2).

In the current study, under alkalinity conditions during the cropping season, the concentration of K^+^ and N in the soil significantly decreased, whereas Na^+^ and P concentration significantly increased. The concentration of Na^+^ in the soil, during the cropping season, increased significantly by 35% and 51% at both levels of alkalinity T1, and T2, respectively, over the control soil. On the other hand, the alkaline soils inoculated with AMF (T1 + AMF and T2 + AMF) decreased Na^+^ concentration compared with the stressed soil. In contrast, alkalinity stress, during the cropping season, reduced K^+^ concentration by 36.5% and 11% in T1 and T2, respectively, compared to the control soil. Inoculation with AMF under unstressed and stressed conditions (T0 + AMF, T1 + AMF and T2 + AMF) significantly reduced K^+^ concentration by 38.1%, 46.8%, and 46.9%, respectively, compared to the control soil (Table 2).

In this experiment, alkalinity stress increased P concentration in the soil during the cropping season with T1 and T2 treatments to 2.8 and 6.3 folds the concentration of the control soil, respectively, whereas AMF inoculation under unstressed and stressed conditions (T0 + AMF, T1 + AMF and T2 + AMF) increased P concentration to 5, 4.3, and 3.4 folds the concentration of control soil, respectively (Table 2). Moreover, N concentration in the soil during the cropping season gradually decreased by 7.14% and 53.6% in T1, and T2, respectively, compared with the control soil. However, inoculation with AMF under both unstressed and stressed conditions (T0 + AMF and T1 + AMF) had negative effects on soil nitrogen concentration which decreased by 50% and 19.6%, respectively; whereas, nitrogen concentration markedly increased by 21%, in T2 + AMF over the control soil (Table 2).

### Mineral composition in plant

The highest alkalinity level (T2) significantly increased Na^+^ concentration in shoots and roots to 101% and 77%, respectively, over the control plants. Inoculation with AMF at the highest level of alkalinity (T2 + AMF) significantly increased Na^+^ concentration by 13.46% and 47.6% in shoots and roots, respectively, over the control plants (Table 3).

**Table 3:** Impact of mycorrhiza inoculation (AMF) and different alkalinity levels (T1, T2) either under stressed and unstressed on minerals content in plants of the wheat cultivar. The data are means ± SD (*n* = 3). Different letters, capital for shoots and small for roots, indicate statistically significant differences according to Tukey’s test *(p* ≤ 0.05)

Treatments	Na^+^		K^+^		P		N	
			**Concentrations (mg/g DW)**					
	**Shoot**	**Root**	**Shoot**	**Root**	**Shoot**	**Root**	**Shoot**	**Root**
**Cont**	5.2 ± 0.14^A^	10.5 ± 0.3^a^	22.7 ± 0.5^D^	7.5 ± 0.13^c^	0.34 ± 0.01^D^	0.17 ± 0.005^b^	0.09 ± 0.0004^F^	0.04 ± 0.0029^c^
**T1**	5.75 ± 0.43^A^	16.2 ± 1.1^c^	21.4 ± 0.9^C^	7.1 ± 0.08^c^	0.16 ± 0.01^B^	0.14 ± 0.004^ab^	0.06 ± 0.0003^D^	0.04 ± 0.0007^c^
**T2**	10.5 ± 0.86 ^B^	18.6 ± 0.3^d^	21.2 ± 0.4^C^	5.9 ± 0.09^b^	0.14 ± 0.006^B^	0.11 ± 0.003^a^	0.05 ± 0.0006^C^	0.03 ± 0.0014^b^
**T0 + AMF**	4.5 ± 0.28^A^	8.50 ± 0.3^a^	18.4 ± 0.1^B^	6.6 ± 0.5^b^	0.26 ± 0.007^C^	0.27 ± 0.18^c^	0.04 ± 0.0004^B^	0.04 ± 0.0005^c^
**T1 + AMF**	5.0 ± 0.14^A^	13.5 ± 0.3^b^	14.0 ± 0.3^A^	6.2 ± 0.1^b^	0.08 ± 0.003^A^	0.32 ± 0.15^d^	0.03 ± 0.0006^A^	0.02 ± 0.0009^a^
**T2 + AMF**	5.9 ± 0.07^A^	15.5 ± 0.3^bc^	18.0 ± 0.4^B^	4.7 ± 0.1^a^	0.16 ± 0.002^B^	0.14 ± 0.0002^ab^	0.07 ± 0.0046^E^	0.03 ± 0.0005^b^

It was observed that K^+^ concentration in roots was more affected than that of the shoots in alkaline stressed plants, which exhibited a noticeable gradual decline by 21.33% in T2 compared with the control plants. On the other hand, inoculation of the soil with mycorrhiza under unstressed conditions (T0 + AMF) reduced K^+^ concentration in the shoots and roots of plants by 19% and 12%, respectively. Besides, the previously mentioned conditions under both levels of alkalinity (T1 + AMF and T2 + AMF) had negative effects on the K^+^ concentration of shoots and roots compared with the control plants (Table 3).

  Phosphorus concentration in the shoots and roots of stressed plants at the higher level of alkalinity was declined significantly by 58.8%, and 35.2% compared to the control plants. AMF inoculated plants exhibited a significant reduction in P concentration of shoots but their concentration in roots had enhancement effects under unstressed and stressed conditions (Table 3).

Similarly, shoots and roots of alkaline stressed plants exhibited a significant inhibition in N concentration, especially in T2. Generally, AMF inoculation under unstressed and stressed conditions reduced significantly the N concentration in both shoots and roots compared with the control plants (Table 3).

### Mycorrhiza colonization

**Fig. 4:**
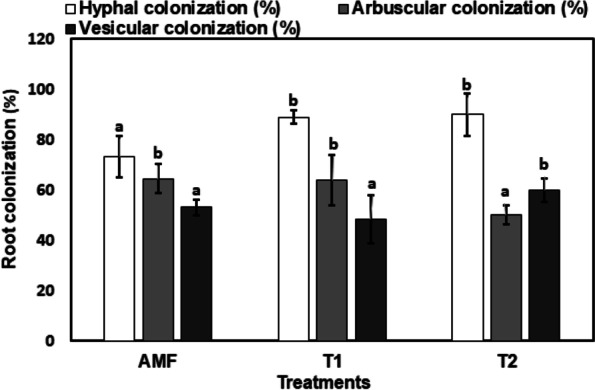
Impact of Mycorrhiza inoculation (AMF) and alkalinity levels (T1, T2) on arbuscular colonization; vesicular colonization and hyphal colonization. The data are means ± SD (*n* = 3). Different letters indicate statistically significant differences according to Tukey’s test (*p* ≤ 0.05)

The results presented in Fig. 4 revealed that hyphal colonization significantly increased with alkaline stress. Meanwhile, the highest value of arbuscular colonization in wheat plants inoculated with *A. laevis*, *F. geosporum*, *F. mosseae*, *C. armeniaca* was found in the control mycorrhizal plants, and the lowest value was recorded in stressed plants with 74 mM NaHCO_3_ (T2). The vesicular colonization of wheat plants ranged from 48.5% to 53.15%, of which the highest value was recorded in plants grown under a high level of alkalinity (T).

### Principal component analysis

**Fig. 5:**
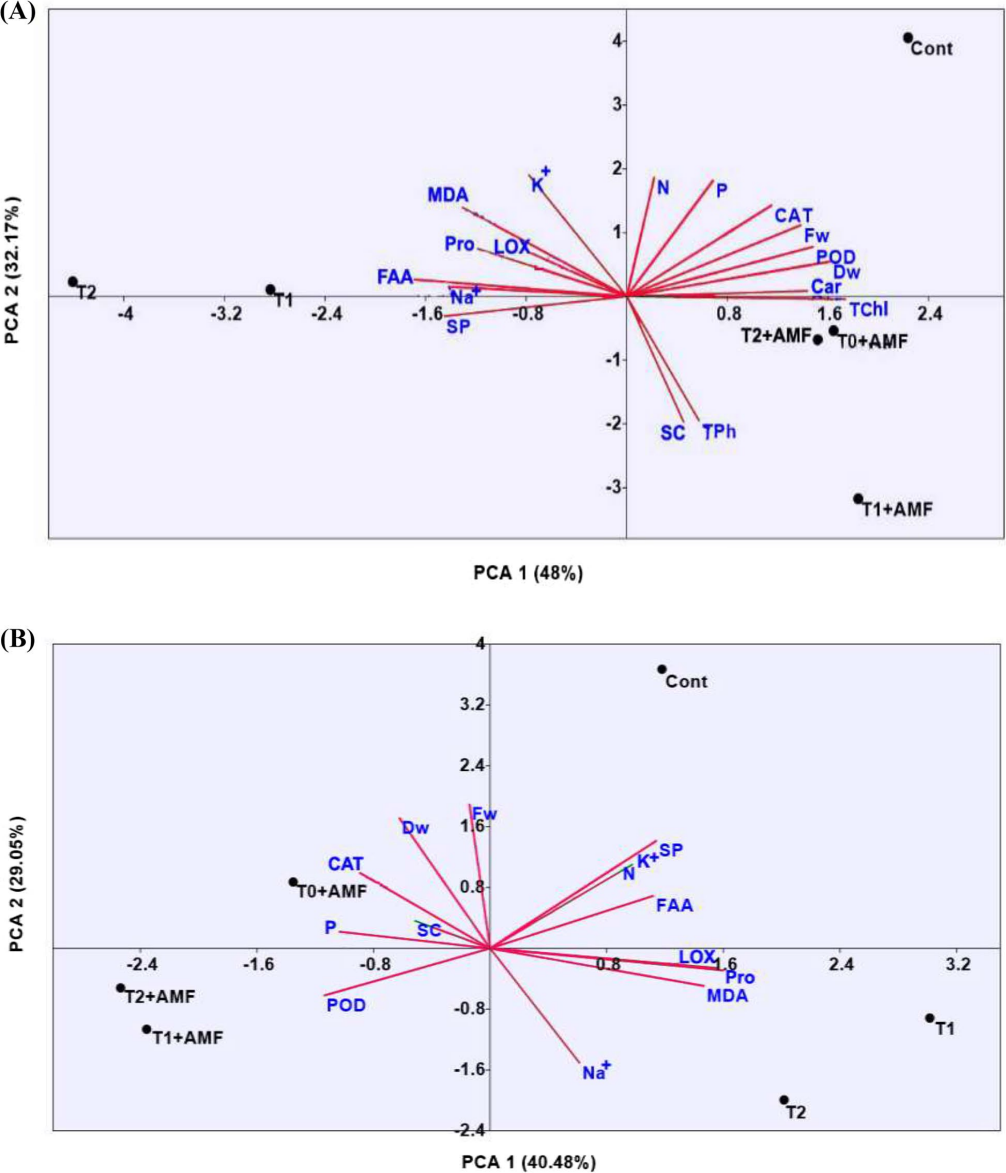
Principal component analysis (PCA) of the studied parameters in shoot and root (**A**, **B**) of different treatments in wheat plant under alkaline and non-alkaline conditions. Cont, Control; (T1 and T2), Alkalinity stress levels; T0 + AMF, Arbuscular mycorrhizal fungi under unstressed condition; T1 + AMF and T2 + AMF, Arbuscular mycorrhizal fungi under stressed conditions; Fw and Dw, Fresh and Dry weight; TChl, Total chlorophylls; Car, Carotenoids; CAT, Catalase; POD, Peroxidase; P, Phosphorous; N, Nitrogen; Na^+^, Sodium; K^+^, Potassium; TPh, Total phenolics; LOX, Lipooxygenase; MDA,Lipid peroxidation; SC, Soluble carbohydrates; SP, Soluble protein; FAA, free amino acids; Pro, Proline

Principal component analysis (PCA) accounted for all the parameters of the different treatments. Two main components accounted for 80.17% of the variability observed in the data of shoots, with 48% for PC1 and 32.17% for PC2 (Fig. 5A). Under control conditions, PCA indicated positive correlations among the growth (Fw and Dw), photosynthetic pigments (TChl and Car), antioxidant enzymes (CAT and POD), and nutrients (N and P).

However, under alkalinity stress (T1 and T2), PCA exhibited a negative impact on these parameters and had positive correlations with primary metabolites (Pro, FAA, and SP), MDA, LOX, and nutrients (Na^+ ^and K^+^). On the other hand, inoculation with AMF under unstressed and stressed conditions (T0 + AMF, T1 + AMF and T2 + AMF) could mitigate these negative effects and have positive impacts on SC and TPh.

In the roots, PCA accounted for 69.53% of the variability observed in the data, with 40.48% for PC1 and 29.05% for PC2 (Fig. 5B). PCA indicated that there were positive correlations among the primary metabolites (FAA and SP) and nutrients (N and K^+^) under the control conditions. Under alkalinity stress conditions (T1 and T2), PCA demonstrated a negative impact on these parameters while under the stressed conditions there were positive correlations with Pro, MDA, LOX, and Na^+^.

PCA also confirmed that inoculation with AMF under unstressed conditions (T0 + AMF) could alleviate these negative effects on these parameters and had positive impacts on the growth (Fw and Dw), SC, antioxidant enzyme (CAT), and P, while AMF inoculation under stressed conditions (T1 + AMF and T2 + AMF) had appositive correlation only with POD.

## Discussion

In both biotic and abiotic environments, AMF increased plant growth, photosynthesis, and stress tolerance [47–49], although the regulatory mechanisms underlying AMF-mediated resistance to alkalinity stress have not been sufficiently explained. In the current study, we observed the effects of mycorrhizal treatment on caryopses germination, plant growth parameters, redox state, plant metabolism, and caryopses yield under alkalinity. Although it is generally accepted that AMF colonization promotes plant growth, the physicochemical condition of the soil may have a direct impact on the plant-fungus symbiotic interaction [50]. A recent study found that mycorrhiza colonization of wheat plants contributes to nutrient acquisition balance, which regulates Na^+^ and Cl^−^ uptake, diminishes membrane permeability and proline, and enhances photosynthetic pigments, leading to wheat plant growth and grain yield improvement during salt stress [51].

  Seed germination is an important stage in a plant's life cycle that is influenced by both abiotic and biotic factors. It begins with the uptake of water by the quiescent dry seed and ends with the radicle emersion through the seed covering layers [52]. In this experiment, it was reported that wheat germination percentage decreased with alkalinity stress. Similar results were found by [47, 48] on seed germination of *Panicum virgatum* and *Triticum aestivum*. Recently, the use of fungal inoculation during seed sowing has been shown to improve crop seed germination and seedling growth [49]. In the current study, mycorrhizal inoculation corroborated the germination percentage in the non-stressed and alkalinity stressed plants. During seed germination, the symbiotically associated fungi degrade cuticle cellulose, providing available carbon, and phytohormones such as gibberellins and indole acetic acid [53]. It was demonstrated that in the first week AMF invasion during the imbibition stage of water throughout the testa begins the association between *Dendrobium* seeds and their symbionts. Hyphae invading cortical cells and forming the peloton enter through the posterior end of the embryo suspensor cell. Pelotons are coiled intracellular hyphae inside cortical cells that contain accumulated organic components like protein, glycogen and fat as a result of nutrition absorption from the soil [54].

  It has been found that the two levels of alkalinity inhibited growth metrics such as the fresh and dry biomass of shoots and roots at the vegetative stage of tested wheat in this study which is in line with the results of [55]. Decreased yield in plants under virtue stress is not a direct response per se. ROS scavenging, photosynthesis, and metabolic changes are all affected when plants are subjected to any kind of stress, which collectively leads to an adjustment in the growth rate as an adaptive response for survival. Recently, some researchers found that the stem diameter, plant height, and dry weight of the roots and shoots of watermelon seedlings were all positively influenced by AMF under salinity-alkalinity stress [6]. The large hyphal network of AMF in the soil absorbs more nutrients, transports nutrients into the fungal intra-radical mycelium, and releases nutrients at arbuscules, permitting roots to absorb more nutrients and water [56].

  Also, the present study demonstrated that alkalinity stress inhibited the synthesis of total chlorophylls and carotenoids of wheat. Previously, it was estimated that *vinca* plants irrigated with water containing 6.8 mM NaHCO_3_ had a 10% drop in chlorophyll concentration [57]. Decreasing Chl. b and carotenoids by increasing salinity/alkalinity stress were also found by [58]; and they ascribed this behavior to the metabolic disorders caused by some ions (e.g., Na^+^) under salt-alkali stress conditions, inhibition of proteinase activity, and change the chlorophyll concentration in leaves. Abiotic stresses cause a decline in the activity of enzymes that regulate chlorophyll biosynthesis and an increase in chlorophyllase activity resulting in chlorophyll degradation [59].

  Mycorrhiza inoculation promoted the synthesis of total chlorophylls and carotenoids in stressed and non-stressed plants, according to the current findings. Many studies found that mycorrhiza increases the photosynthetic pigments in the host plants [58]. Mycorrhizal inoculation enhanced chlorophyll concentration and induced higher uptake of N and Mg but inhibited Na-transport even under saline conditions [60]. It is reasonable to highly probable that AMF inoculation reduced chlorophyllase activity while increasing the expression of chlorophyll biosynthetic genes, leading to increase pigment synthesis [61].

  Under alkalinity and salinity stresses, an ionic imbalance occurs, disrupting the regulation of the osmotic balance. As a result, a variety of small-molecule organic substances were synthesized and stored by plant cells such as proline, soluble proteins, betaine, sugar, polyols, and polyamines to regulate the water potential [62]. Proline accumulation is an adaptive mechanism against salt stress [63]. In the present study, proline exhibited the highest concentration in wheat plants grown on alkalinized soils. Also, It was found that under salinity-alkalinity stresses the increase in proline concentration indicated an efficient plant stress response at the metabolic level [6]. Mycorrhiza inoculation inhibited the accumulation of proline in leaves and roots of tested wheat. However, microbe inoculation can minimize environmental stress levels in plants that did not have to synthesize excessive amounts of osmotic adjustment or antioxidant substances to cope with stress [64]. In the present study, the soluble protein in leaves increased with rising alkalinity levels; however, mycorrhizal inoculation decreased its concentration in wheat roots under unstressed and stressed conditions. It has been demonstrated that total soluble protein concentration in *Jatropha* leaf showed a linear increase with increasing Na_2_CO_3_ concentrations (from 0.1% to 0.4%) as a mechanism to maintain the osmotic balance and leaf water status at high alkalinity [65]. Any modifications in protein concentration may lead to accumulation or depletion of certain metabolites resulting in an imbalance in the levels of a relatively small set of cellular proteins, which could increase/decrease, appear/disappear after stress treatment [66]. Mycorrhizal inoculated wheat had a higher protein concentration in the roots than non-mycorrhizal plants grown in saline soil [67]. In the present study, soluble sugar accumulation in wheat leaves and roots increased at the highest level of alkalinity. Under the same alkalinity level, inoculation with mycorrhiza additively enhanced sugar accumulation in leaves. The increase in soluble sugar may be a natural response to offer protection against damage by reactive oxygen species *via* reinforcing the antioxidant system [68]. The high levels of sugars in mycorrhizal plants may be due to an increase in photosynthetic capacity [69]. At moderate and severe alkalinity, plants accumulate more carbohydrates and significantly increase their ROS and MDA concentrations [70]. The role of free amino acids (FAAs) in plant stress response has been attributed to osmotic adjustment, ion transport, and the re-assimilation of nitrogen released from other biological processes as available sources of carbon and nitrogen [71]. In the current study, the concentration of free amino acids increased in wheat leaves in all alkalinity treatments but decreased at most in roots. The current results are in agreement with the previous study on jojoba and sunflower which found that the levels of amino acids in the leaves increased by saline or alkaline stresses. They attributed this to de novo synthesis and/or degradation of proteins [72]. The concentration of amino acids in the shoots and roots of the mycorrhizal inoculated studied wheat decreased either in stressed or unstressed plants compared to those that were non-inoculated. Plants colonized by AMF have lower levels of metabolites related to amino acid metabolism compared to higher levels. Furthermore, the detrimental effect of mycorrhization on amino acid metabolism may indicate an imbalance in the supply of inorganic phosphorus and nitrogen to inoculated plants when compared to the control ones [73].

  MDA is a result of the degradation of polyunsaturated membrane fatty acids and is used to assess the severity of oxidative damage [11]. In comparison to the controls, alkaline enhanced lipid peroxidation in wheat, but it decreased in mycorrhizal inoculated plants. This is consistent with the findings of [2], who discovered that MDA damages cell membranes, increases significantly in response to salinity-alkalinity stress in the watermelon plant, and decreases significantly after AMF treatment when compared to the controls. Also, [64] stated that mycorrhizal inoculation inhibited the synthesis of MDA and consequently alleviated oxidative damage and ROS accumulation.

  LOX initiated the plant oxylipin pathway and developed jasmonic acid (JA), which participates in the biotic and abiotic stress defense response [74]. As a result, LOX activity increased and largely depends on inducing agents as well as the plant genotype and physiological conditions [75]. The present study revealed that alkalinity stress mostly promoted LOX activity in wheat, while plants inoculated with mycorrhiza significantly lowered LOX activity in parts of stressed and unstressed plants. This agreed with the work of [76] who reported that salinity stimulated LOX activity in the tomato plant and that increasing LOX activity leads to an increase in lipid peroxidation at the alkalinity stress.

  To detoxify ROS for alleviating oxidative stress damage, plants often employ antioxidants, including enzymatic and non-enzymatic mechanisms [77]. Catalase (CAT), one of the enzymatic antioxidants, catalyzes the detoxification of H_2_O_2_ to H_2_O and O_2_ [78]. In the current study, alkalinity decreased catalase and peroxidase activity in leaves, while these enzymes increased mostly in roots. This increase indicates that high absorption of micronutrients from the soil activates these enzymes in roots. Modifications in the activity of CAT, POX, and ROS concentration were reported in wheat plants in field and laboratory conditions [60]. According to the study of [62] the sorghum seedlings exhibited sharply increased POD activity with increasing alkalinity, especially at a high salt level. In the current study, mycorrhizal inoculation in alkaline and unstressed soils increased the catalase and peroxidase activity of wheat over the alkalinity treatments. This is consistent with [79] who observed an increase in the activity of antioxidant enzymes of fungal treated plants, which may be attributed to the contribution of hyphal transport of slowly diffusing micronutrient ions such as Zn and Cu, which serve as co-factors for these enzymes.

  Some authors revealed that under abiotic stress AMF inoculation significantly boosted the enzyme activity and gene expressions involved in ROS homeostasis, resulting in improved oxidative stress tolerance in the host plant [80]. Then, ROS does not cause cellular harm while also integrating multiple developmental processes and cell differentiation [61].

  Non-enzymatic antioxidants are involved in the declining accumulation of ROS [81]. The phenolic compounds act as antioxidants in different plant tissues by mitigating oxidative stress and scavenging the ROS [82]. The concentration of total phenolics in the studied wheat increased by alkalinity, but mycorrhizal inoculation highly promoted its accumulation under stressed and non-stressed conditions. This agreed with a previous study which indicated an increase in total phenol in cucumber plants exposed to salinity and AMF treatments [56]. Additionally, [83] demonstrated that a greater accumulation of phenols in AMF-inoculated plants confers stress tolerance. The colonization of fungi increased the activity of chalcone synthase and chalcone isomerase enzymes that are involved in the synthesis of phenylalanine ammonia-lyase catalyzing the deamination of phenylalanine which is important for the regulating stage in the formation of phenolic compounds [84].

  The findings of this study revealed that alkalinity stress had a significant impact on wheat caryopses yield parameters, while AMF inoculation significantly improved them. This agreed with the work of [85] who found that increasing pH from 7.2 to 9.1 and 9.4 decreased the caryopses yield of wheat to 69.3% and 95.7%, respectively, at stages, which delayed the flowering of panicles.

  In the current study alkalization with NaHCO_3_ increases the electrical conductivity (EC) and pH values in the soil, while inoculation with AMF decreased both EC and pH in the treated soil. These results are consistent with [65] who found that with the increase of Na_2_CO_3_ concentration, soil pH increased from 7.5 to 11. According to [86], AMF colonization can alter the concentrations of organic acids in root exudates, lowering soil pH and electrical conductivity. In this study, Na^+^ concentration in the soils was increased by the addition of NaHCO_3_ while K^+^ concentration in soil at harvesting was decreased in all treatments. A similar situation was found in wheat shoots and roots, where the concentration of Na^+^ was increased, while K^+^ was decreased by alkalinity. By increasing soil pH, the availability of most nutrients like K^+^ decreases. Additionally, in sodic soils, the competition of Na^+^ with other essential cations limits their availability to plants [87].

  AMF has been demonstrated to have a beneficial effect on the absorption and selection of mineral nutrients by plants through their numerous extraradical mycorrhizal hyphae distributed throughout the soil [88, 89]. Alkalinity stress was found to increase soil P concentration, whereas soil inoculated with mycorrhiza under the same conditions had the opposite effect [90]. Likewise, P availability in alkaline soils can be increased by adjusting the pH of the rhizosphere of some plants [91].

  In the present study, there was a negative relationship between soil nitrogen concentration and alkalinity levels. AMF inoculation at a high level of alkalinity increased N concentration. A previous study showed the negative relationship between N mineralization and ammonification with EC (electrical conductivity), SAR (sodium adsorption ratio), and ESP (exchangeable sodium percentage). This indicates that an increase in salinity and sodicity is injurious to most of the microbes, and in that situation, less metabolically efficient soil microbes mediate soil processes including mineralization of C, N, P, and S [92].

  The present study revealed that the shoots of wheat grown on alkalinized soils with AMF inoculation decreased Na^+^ concentration compared to non-inoculated plants. This is in agreement with [67] who found that mycorrhizal fungi could reduce salt stress by preventing Na^+^ uptake and translocation to shoot tissues. In the previous study, two explanations are possible. First, AMF-inoculated plants were able to keep Na^+^ inside intraradical fungal hyphae and root cell vacuoles, limiting the harmful transport ions from roots to shoots [23]. Second, AMF symbiosis may cause the expression of Na^+^/K^+^ transporters, which allow plants to sequester Na^+^ in vacuoles and limit Na^+^ entrance into root systems [93].

  Our results showed that alkalinity stress had negative effects on the accumulation of K^+^ concentration in stressed wheat plants. This is consistent with the previous experiment [8] which demonstrated that the application of 5 mM NaHCO_3_ to soil impairment shoots K^+^ concentration in two genotypes of *Brassica juncea* below adequate concentration. Similarly, inoculating the soil with mycorrhiza under both unstressed and stressed conditions suppressed the accumulation of K^+^ in the shoots and roots of plants. This finding is consistent with [94] who indicated that increasing the percentage of mycorrhizal colonization reduced the potassium content of roots and shoots.

  Our results indicated that AMF inoculated plants exhibited a significant reduction in P concentration of shoots but their concentration in roots had enhancement effects under unstressed and stressed conditions. AMF inoculation that decreased shoots phosphorus concentration may be due to the decreased translocation of P from roots to shoots. Accordingly, [95] reported that under high P (HvPHT1;6 as a barely P transporter) function at a low-affinity range. A previous study indicated an increase in phosphorus concentration in the leaves and roots of *Zelkova serrata* seedlings inoculated with two strains of mycorrhiza under salt stress compared to non-mycorrhizal seedlings. These findings were consistent with numerous previous studies that claimed AMF can improve P absorption and raise its concentration in plants with salt stress conditions [96]. The study of [97] reported that plants grown in alkaline soil used a mechanism to produce mycorrhiza through increased root and hairy root growth, which helps in the mobile building of phosphorus and also allows external mycorrhiza fungal chains to sneak H^+^ ions into the soil, resulting in an adjusted pH. There is a significant ratio of mineral phosphorus that can be “biologically fixed” by mycorrhiza when soil phosphorus levels are low.

  The results of the current study showed that N concentration in the shoots and roots of stressed plants gradually decreased, while those inoculated with AMF under the highest level of alkalinity exhibited a significant increase. This finding agreed with the previous study which showed that N content in stressed plants exhibited a decline markedly more than in mycorrhizal inoculated ones [98]. Furthermore, improved N uptake by AMF inoculation may help to decrease the harmful effects of Na^+^ ions by regulating their uptake and indirectly maintaining the chlorophyll content of the plant [99].

  In the present study, high pH (alkali stress) significantly increased hyphal and vesicular colonization [25, 100], while decreasing arbuscular colonization [101], and this, concomitantly, inhibited mycorrhizal growth. The formation of arbuscular in host plants increases the surface area of the plant-fungus interface and magnifies the efficacy of resource exchange. The main factor was that alkali stress reduced spore germination due to high pH stress [101]. Mycorrhizal colonization is also affected by the pH of the rhizosphere, as well as the plant and fungal species participating in such association.

## Conclusion

According to these results, we may conclude that alkalinity stress had highly negative effects on germination percentage, growth parameters such as fresh, dry weight, total chlorophylls, carotenoids and yield of the wheat plant. However, under the previously mentioned conditions, some metabolites, lipid peroxidation, antioxidants enzymes and phenolic compounds increased. AMF inoculation reduced the pH of alkaline soil, increasing P availability for plants compared to alkaline soil alone. Furthermore, compared to alkalinity-stressed plants alone, AMF inoculation lowered Na^+^ concentration in alkaline soil, resulting in lower Na^+^ concentration in shoots and roots. Moreover, AMF inoculation induces the scavenging of free radicles by inhibiting the synthesis of MDA and promotes the activity of some enzymatic and non-enzymatic antioxidant as catalase, peroxidase and total phenolic. The findings showed that the benefits of mycorrhizal symbiosis extend beyond host nutrition and growth stimulation, and they can assist in understanding the multiple effects of mycorrhiza under various conditions, which is essential for their use in agriculture. This technique included a consortium of arbuscular mycorrhizal fungi (*A. laevis, F. geosporum, F. mosseae,* and *C. armeniaca*.), which enhance plant tolerance and increase wheat productivity under alkalinity conditions.

## Data Availability

All data generated or analyzed during this study are included in this published article and its supplementary information file is not publicly available due to its proprietary nature. Supporting data cannot be made openly available but are available from the corresponding author on reasonable request.
